# Botanical drug preparations and related East Asian medicine interventions for central and idiopathic central precocious puberty: an umbrella review of systematic reviews and meta-analyses

**DOI:** 10.3389/fphar.2026.1697393

**Published:** 2026-08-19

**Authors:** Yuanyuan He, Shixin Zhuang, Akzhan M. Madenbayeva, Nadiar M. Mussin, Amin Tamadon, Jian Yu

**Affiliations:** 1 Traditional Chinese Medicine Department, Children’s Hospital of Fudan University, Shanghai, China; 2 Respiratory Department, Children’s Hospital of Fudan University, Shanghai, China; 3 Department of Internal Diseases No 1, West Kazakhstan Marat Ospanov Medical University, Aktobe, Kazakhstan; 4 Department of General Surgery, West Kazakhstan Marat Ospanov Medical University, Aktobe, Kazakhstan; 5 Department of Natural Sciences, West Kazakhstan Marat Ospanov Medical University, Aktobe, Kazakhstan

**Keywords:** gonadotropin-releasing hormone, medicine, Chinese traditional, meta-analysis as topic, phytotherapy, precocious puberty, central

## Abstract

**Background:**

Central precocious puberty (CPP), particularly idiopathic CPP (ICPP), is characterized by the premature activation of the hypothalamic–pituitary–gonadal axis. Gonadotropin-releasing hormone agonists (GnRHas) are established biomedical treatments, but treatment burden, cost, and incomplete long-term outcome data have encouraged interest in adjunctive botanical drug preparations used in East Asian medicine. However, these preparations differ substantially in composition, dose, comparator, and reporting quality.

**Objectives:**

The aim of this study is to synthesize systematic reviews (SRs) and meta-analyses (MAs) evaluating botanical drug preparations and related East Asian medicine interventions for CPP/ICPP, appraise methodological quality and certainty, quantify the overlap of primary trials, and identify evidence gaps.

**Methods:**

Eligible studies included systematic reviews/meta-analyses of randomized trials in children with CPP/ICPP. Databases were searched from inception to September 2025. Evidence was narratively synthesized by formulation family, comparator, and outcome. Review quality was assessed using AMSTAR-2, risk of bias using ROBIS, certainty using GRADE domains where feasible, and primary-trial overlap using the corrected covered area.

**Results:**

Four systematic reviews/meta-analyses were included. Favorable signals were reported mainly for Zhibai Dihuang-based preparations, Dabuyin-based preparations, Nourishing Yin–Purging Fire regimens, and botanical drug preparations combined with GnRHas. Outcomes included clinical response, uterine/ovarian volume, gonadotropin levels, estradiol levels, and bone age index. However, interpretation was limited by heterogeneous formulations, variable comparators, an unclear or high risk of bias in the primary trials, incomplete formulation reporting, possible publication/language bias, and overlap of primary studies. Adverse events were incompletely defined and may have been under-reported; long-term pediatric safety was rarely assessed.

**Conclusion:**

Current evidence suggests a possible adjunctive benefit of selected botanical drug preparations, particularly in combination with GnRHas, for CPP/ICPP. However, the certainty of the evidence remains low because of methodological weaknesses, heterogeneous interventions, single-country evidence, incomplete safety reporting, and limited long-term outcome data. High-quality multicenter trials with standardized botanical authentication, dosing, outcome definitions, adverse-event surveillance, and long-term follow-up are needed before routine clinical integration can be recommended.

## 1 Introduction

Precocious puberty is classically defined as the onset of secondary sexual characteristics before 8 years in girls and before 9 years in boys (Mucaria et al., 2021). When early pubertal activation reflects premature stimulation of the hypothalamic–pituitary–gonadal axis without an identifiable cause, the condition is termed idiopathic central precocious puberty (ICPP) (Srilanchakon et al., 2025). Beyond its immediate clinical manifestations, central precocious puberty (CPP)/ICPP is associated with accelerated skeletal maturation, potential compromise of final adult height, and psychosocial challenges for affected children and families (Knific et al., 2022).

Conventional therapy centers on gonadotropin-releasing hormone agonists (GnRHas), which suppress gonadotropin secretion to halt pubertal progression and slow bone age advancement (Shi et al., 2022). Although effective for many patients, GnRHa therapy can be limited by injection burden, treatment costs, and adverse effects and may not uniformly normalize growth velocity or optimize adult height—especially in children who present later with advanced bone age (Cho et al., 2023). Other agents, such as megestrol acetate, have been used in selected contexts to influence uterine volume or symptoms, but pediatric safety and long-term effectiveness remain uncertain (Shim et al., 2024). Against this backdrop, there is growing interest in traditional Chinese medicine (TCM) and Chinese herbal medicine (CHM)—including formula families such as Zhibai Dihuang and Dabuyin and broader Nourishing Yin–Purging Fire (NYPF) strategies—either as monotherapy or more commonly as adjuvants to GnRHa (Ma et al., 2023). Reports suggest potential benefits on pelvic ultrasound indices, sex hormone suppression, and bone age indices, often with favorable tolerability (Talarico et al., 2021).

Over the past decade, multiple systematic reviews (SRs) and meta-analyses (MAs)—including conventional pairwise syntheses and network meta-analyses—have examined CHM/TCM for CPP/ICPP (Cheng and Wan, 2025). However, their conclusions vary due to differences in eligibility criteria, herbal formulations, dosing, comparators, outcome definitions, and risk-of-bias appraisal (Lee et al., 2020). In addition, primary randomized trials are frequently small, single-center, and methodologically heterogeneous, and several reviews overlap in the trials they include (Brantner et al., 2024). Consequently, clinicians and policymakers face uncertainty about the consistency, magnitude, and certainty of CHM effects, the incremental value of CHM + GnRHa over GnRHa alone, and the safety profile across formulations (Lee et al., 2020). An umbrella review—a review of systematic reviews/meta-analyses—can address these gaps by synthesizing the highest-level evidence, mapping overlap, comparing findings across reviews, and appraising methodological quality, thereby providing a clear, practice-oriented summary (Ch et al., 2023).

In this umbrella review, we aim to synthesize and critically appraise existing systematic reviews and meta-analyses evaluating the efficacy and safety of TCM/CHM [and related Korean medicine (KM) interventions] for children with CPP/ICPP. Specifically, we (i) summarize the effects on clinical efficacy, secondary sexual characteristics (uterine/ovarian volume and breast development), sex hormones [follicle-stimulating hormone (FSH), luteinizing hormone (LH), and estradiol (E2)], and bone age index; (ii) characterize the safety profile (adverse events); (iii) examine subgroup signals; (iv) assess methodological quality of included reviews and overlap of primary trials; and (v) identify evidence gaps and priorities for future research.

## 2 Methods

### 2.1 Protocol and registration

This umbrella review was conducted in accordance with the PRISMA 2020 statement (Page et al., 2021), with methodological guidance for umbrella reviews taken from the Joanna Briggs Institute (JBI) approach (Aromataris et al., 2015). The review protocol was prospectively registered with OSF (https://doi.org/10.17605/OSF.IO/2NJAV).

### 2.2 Eligibility criteria

Studies were eligible if they met the following criteria:

Population: Children diagnosed with CPP or ICPP.

Interventions: We included two intervention categories: (i) botanical drug preparations used in TCM, CHM, or KM, including decoctions, pills, granules, or formula families such as Zhibai Dihuang, Dabuyin, and NYPF regimens; and (ii) related non-botanical East Asian medicine modalities when assessed in eligible reviews, including auricular plaster therapy, acupoint therapy, or moxibustion, alone or as adjuncts to biomedical therapy, particularly GnRHa. Botanical and non-botanical modalities were extracted and interpreted separately.

Comparators: GnRHa, placebo, megestrol acetate, nutritional supplements, or no treatment.

Study types: SRs and MAs of randomized controlled trials (RCTs).

Outcomes: Clinical efficacy, secondary sexual characteristics (uterine/ovarian volume and breast development), sex hormone levels (FSH, LH, and E2), bone age index (BAI), growth rate, and adverse events.

Language restrictions: Reviews published in English.

### 2.3 Information sources and search strategy

A comprehensive search of the following databases was performed: PubMed/MEDLINE, Web of Science, and Scopus. Because this was an umbrella review, the unit of inclusion was the systematic review/meta-analysis rather than the individual primary RCT. The search was restricted to English-language SRs/MAs indexed in PubMed/MEDLINE, Web of Science, and Scopus. Chinese and Korean bibliographic databases, including CNKI, Wanfang, VIP/SinoMed, KoreaMed, KMbase, OASIS, KISS, and RISS, were not searched for additional non-English SRs/MAs. This decision was made to ensure consistent full-text assessment, duplicate-study extraction, methodological appraisal, and overlap mapping across reviews available to the review team. We acknowledge this as an important limitation because eligible non-English Chinese or Korean evidence syntheses may have been missed. The search covered all records published from inception to 1 September 2025. In addition, the reference lists of included studies and relevant reviews were hand-searched to identify additional eligible publications. The search strategy is shown in Table 1.

**TABLE 1:** Search strategy.

Concept	Keywords/synonyms	Code
Population/condition	“Precocious puberty” OR “early puberty” OR “central precocious puberty” OR CPP	#1
Intervention	“Traditional Chinese medicine” OR “Chinese herbal medicine” OR “Chinese medicine” OR “herbal medicine” OR “nourishing yin” OR “purging fire” OR “Zhibai Dihuang” OR “Dabuyin” OR “Korean medicine” OR “East Asian medicine”	#2
Study type	Meta-analysis OR “systematic review”	#3
Combined search	#1 AND #2 AND #3	#4
Limits	Language: English; date: no restriction	​

### 2.4 Study selection

Two reviewers independently screened titles, abstracts, and full texts using the eligibility criteria. Discrepancies were resolved by consensus or consultation with a third reviewer. The study selection process was documented according to PRISMA 2020 reporting guidance (Page et al., 2021).

### 2.5 Data extraction

Data were independently extracted by two reviewers using a standardized form. Extracted information included the following: first author and year of publication, country/region of review, number of included studies and participants, interventions and comparators evaluated, outcomes assessed, main findings (effect sizes and direction of effect), and limitations reported by authors.

For botanical drug preparations, we additionally extracted the formula name, pinyin name, reported drug materials, plant part or pharmacopeial drug name, dose and dosage form where available, and whether the original review or primary trial reported species-level composition. Botanical names were checked against Plants of the World Online and Kew’s Medicinal Plant Names Service. Where species-level composition, plant authentication, voucher information, or quality-control methods were not reported in the original evidence source, this was recorded as “not reported” and was not inferred for formulation-specific efficacy conclusions.

To improve transparency and address intervention comparability, the principal botanical drug preparations were extracted at the drug-material level, including formula/preparation name, pinyin drug-material name, taxonomically validated scientific name with authority, family, pharmacopeial drug name or plant part, and whether the composition was explicitly reported in the original evidence source; non-botanical drug materials were identified separately (Supplementary Table S1).

### 2.6 Quality assessment of reviews

The methodological quality of each included SR/MA was independently assessed by two reviewers using AMSTAR-2 (Shea et al., 2017), a 16-domain critical appraisal tool for systematic reviews of healthcare interventions. Overall confidence was reported using only the standard AMSTAR-2 categories: high, moderate, low, or critically low. Hybrid categories such as “low–moderate” or “moderate–high” were not used. Overall judgments were derived from the number and importance of critical and non-critical domain weaknesses, according to the AMSTAR-2 guidance. Disagreements were resolved by consensus or consultation with a third reviewer.

### 2.7 Risk of bias and certainty of evidence

In addition to AMSTAR-2 methodological appraisal (Shea et al., 2017), we assessed risk of bias at the systematic-review level using ROBIS (Whiting et al., 2016). Only one included review reported full GRADE assessments. Therefore, for outcomes covered by that review, we extracted the reported GRADE certainty. For reviews without formal GRADE tables, we did not perform a full *de novo* GRADE assessment from primary-trial data because this umbrella review was conducted at the review level. Instead, we provided an author-derived umbrella-review certainty judgment informed by the GRADE domains (Guyatt et al., 2008), including risk of bias, inconsistency, indirectness, imprecision, publication bias, and overlap of primary trials. These judgments are presented as umbrella-review certainty judgments rather than formal *de novo* GRADE ratings.

### 2.8 Data synthesis

A narrative synthesis was performed because the included reviews differed in formulation composition, comparator, outcome definition, dose, and statistical approach. Findings were stratified by formulation family and comparator: botanical drug preparation plus GnRHa versus GnRHa alone; botanical drug preparation versus GnRHa; botanical drug preparation versus megestrol, placebo/no treatment, or nutritional supplements; and network meta-analysis comparisons. We avoided pooling effect estimates across clinically incompatible comparisons.

Primary-study overlap was assessed using a citation matrix and quantified using corrected covered area (CCA), as described by Pieper et al. (2014). Overall and pairwise CCAs were calculated as follows: CCA = (N − r)/(r × c − r), where N is the total number of primary-study occurrences across reviews, r is the number of unique primary studies, and c is the number of included reviews. CCA was interpreted as slight, moderate, high, or very high overlap using established thresholds (Pieper et al., 2014).

### 2.9 Data visualization and statistical software

All figures were generated using RStudio (R version 4.2.3; RStudio PBC, Boston, MA, United States). Data from the included systematic reviews and meta-analyses were first organized into structured spreadsheets (Excel, Microsoft Corp.) and then imported into RStudio for visualization. For quality assessment, the AMSTAR-2 ratings were transformed into numeric codes (0 = “No/Not reported,” 1 = “Partial,” and 2 = “Yes”) to facilitate plotting. Bubble plots were created in which each bubble represented a review’s rating for an individual AMSTAR-2 domain, with bubble size and color reflecting the assigned score. This was implemented using the ggplot2 package. Stacked proportional bar charts were used to show the overall distribution of AMSTAR-2 ratings across the four reviews. The proportions of “Yes,” “Partial,” and “No/Not reported” were calculated and plotted as percentages.

For overlap analysis, the inclusion of primary studies across reviews was coded in binary form (0 = “not included”; 1 = “included”). Heatmaps displaying study-by-review overlap, created using the pheatmap package. For outcome synthesis, extracted effect directions were coded as follows: ↓ = significant reduction, ↑ = significant increase, and ns = non-significant change. These codes were then tabulated and used to generate summary figures for visual comparison across reviews. All scripts were written using R packages including tidyverse, ggplot2, forcats, pheatmap, and metafor. The code has been archived and is available upon request to ensure transparency and reproducibility.

## 3 Results

### 3.1 Study selection

**FIGURE 1:**
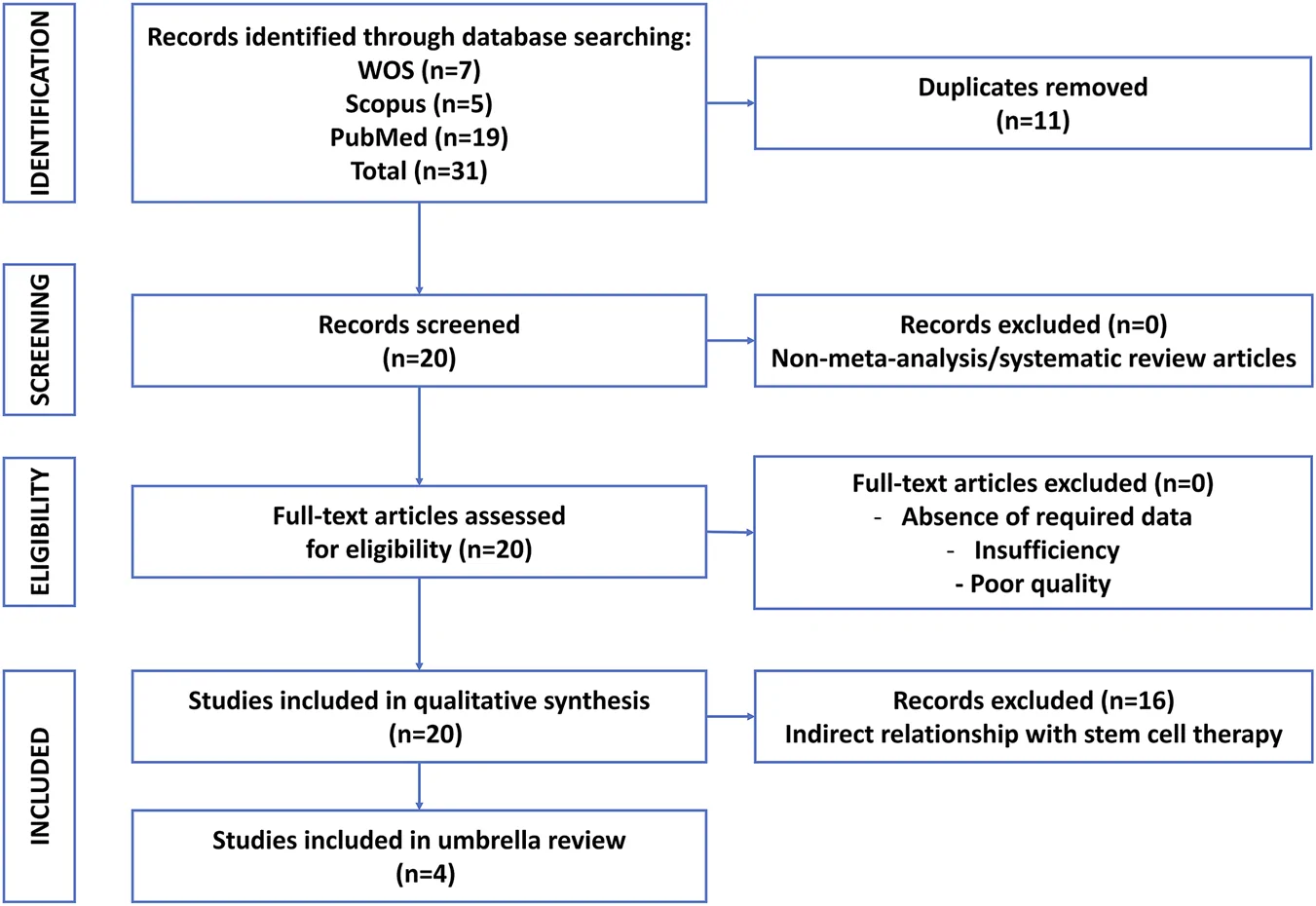
PRISMA flow diagram illustrating the selection process of systematic reviews and meta-analyses included in the umbrella review.

The electronic database search and manual screening identified a total of 31 records. After removal of duplicates, 20 studies remained for title and abstract screening. Of these, 16 articles were excluded for not meeting the eligibility criteria. The full texts of four articles were reviewed, and none were excluded for reasons such as not being randomized controlled trials, not involving traditional Chinese medicine or Korean medicine interventions, or providing insufficient outcome data. Finally, four systematic reviews and meta-analyses met the inclusion criteria and were incorporated into this umbrella review. The selection process is summarized in the PRISMA flow diagram (Figure 1).

### 3.2 Characteristics of included reviews

A total of four systematic reviews and meta-analyses published between 2020 and 2025 were included, encompassing between 9 and 81 RCTs with sample sizes ranging from 650 to more than 2,000 participants (Table 2). All reviews focused on children with ICPP, and most of the primary studies were conducted in China.

**TABLE 2:** Characteristics of included systematic reviews and meta-analyses on botanical drug preparations and related East Asian medicine interventions for CPP/ICPP.

Author, year (reference)	Country/region	No. of studies included	Total sample size	Intervention(s)	Intervention category	Comparator(s)	Outcomes assessed	Main findings	Limitations reported
Cheng and Wan (2025)	China	17 RCTs (1998–2023)	1,590 (811 CHM + GnRHa; 780 GnRHa alone)	CHM (mainly Zhibai Dihuang, Dabuyin, Zhibai Jianghuo, and Ziyin Xiehuo) + GnRHa	Botanical drug preparation	GnRHa alone	Uterine and ovarian volume, bone age index, FSH, LH, E2, and adverse events	CHM + GnRHa ↓UV (SMD −0.97), ↓OV (SMD −0.74), ↓BAI (SMD −1.69), and ↓FSH, LH, and E2. Zhibai Dihuang most effective. No serious AEs; nausea/vomiting most common	High heterogeneity; some studies unclear in hormone measurement timing; all from China; limited long-term outcomes
Lee et al. (2020)	Korea	9 RCTs	n = 650	Herbal medicine (decoctions, pills, and granules); some trials HM ± other modalities	Botanical drug preparation + GnRHa	Megestrol, GnRHa (triptorelin/leuprolide), and no treatment	Response rate; LH, FSH, and E2; uterine/ovarian volume; bone age index (BAI); AEs	HM ↑response rate vs. controls (RR 1.14); HM ↓E2 vs. controls; vs. triptorelin, HM ↓LH and E2; HM ↓BAI and uterine volume vs. controls; AEs low and not ↑ vs. controls. PRISMA and meta-analytic details shown on pp.3–6 and figs/tables on pp.14–22	Small Chinese RCTs; high heterogeneity; unclear/randomization and no blinding/placebo; variable outcome definitions; generalizability limited
Ma et al. (2023)	China	23 RCTs (2001–2023)	2,036 (1,024 NYPF; 1,012 GnRHa)	Nourishing Yin and Purging Fire (NYPF) therapy ± GnRHa	Related non-botanical East Asian medicine modality	GnRHa	Clinical efficacy rate, TCM syndrome scores, uterine and ovarian volume, breast nucleus and follicular diameter, FSH, LH, E2, bone age index, and AEs	NYPF ± GnRHa improved secondary sexual indices, ↓FSH, LH, and E2, ↓BAI, ↑clinical efficacy. Core herb pair: *Zhimu-Huangbai* (quercetin, kaempferol, and β-sitosterol implicated). Fewer AEs vs. GnRHa (esp. vaginal bleeding)	Studies of low quality; unclear randomization; high heterogeneity; all Chinese studies; mechanism findings largely from network pharmacology, not clinical confirmation
Shim et al. (2024)	Korea	81 RCTs	Not reported in total; all children with ICPP	Korean medicine interventions (herbal medicine, auricular plaster, acupoint therapy, moxibustion, and combinations with GnRHa)	Mixed botanical and non-botanical East Asian medicine interventions	GnRHa, other KM, nutritional supplements, and placebo	Growth rate, bone age index, uterine volume, ovarian volume, FSH, LH, E2, and adverse events	HM + GnRHa and HM + auricular plaster therapy ranked the highest for hormone control and pelvic ultrasound indices. Combination therapies generally > monotherapies	All trials Chinese, small-scale; high heterogeneity; many with “some concerns” or high risk of bias; lack of allocation concealment/blinding; no long-term outcomes

The interventions evaluated were primarily herbal medicine (HM) decoctions, pills, or granules, often based on Zhibai Dihuang and Dabuyin formulas. Some reviews investigated broader categories, such as NYPF therapy, or combination approaches that included auricular plaster therapy, acupoint therapy, or moxibustion. Most reviews compared HM or combined regimens against GnRHa (triptorelin and leuprorelin), although additional comparators included megestrol acetate, placebo, nutritional supplements, or no treatment.

Across reviews, the most frequently reported outcomes included clinical efficacy, secondary sexual characteristics (uterine/ovarian volume and breast/follicular diameter), sex hormone levels (FSH, LH, and E2), BAI, growth rate, and adverse events (AEs).

All four reviews reported favorable signals for selected botanical drug preparations or preparation families, particularly when used as adjuncts to GnRHa. However, these findings should not be interpreted as evidence that TCM/CHM, as a broad category, has a uniform pharmacological effect. The included interventions differed in formulation composition, dose, dosage form, comparator, and treatment duration, and several reviews did not provide sufficient species-level or quality-control information for all primary trials. Zhibai Dihuang emerged as the most effective individual formula in several analyses, while the herb pair Zhimu–Huangbai was highlighted as the mechanistic core of NYPF therapy. Combination regimens, including botanical drug preparations plus GnRHas and botanical drug preparations plus auricular plaster therapy, ranked highly in network analyses for some hormone and ultrasound outcomes; however, these rankings should be interpreted cautiously because the interventions, comparators, and trial quality differed substantially. No serious adverse events were reported in the included reviews; however, adverse event definitions, severity grading, active surveillance, and long-term pediatric safety outcomes were inconsistently reported.

Despite consistent signals of benefit, all reviews noted substantial methodological weaknesses in the primary RCTs, including small sample sizes, inadequate randomization, lack of blinding or placebo control, and high heterogeneity. Furthermore, all RCTs were conducted in China, limiting generalizability. Mechanistic evidence was preliminary and not yet clinically validated. Long-term outcomes such as final adult height were rarely assessed.

### 3.3 Quality of reviews

**FIGURE 2:**
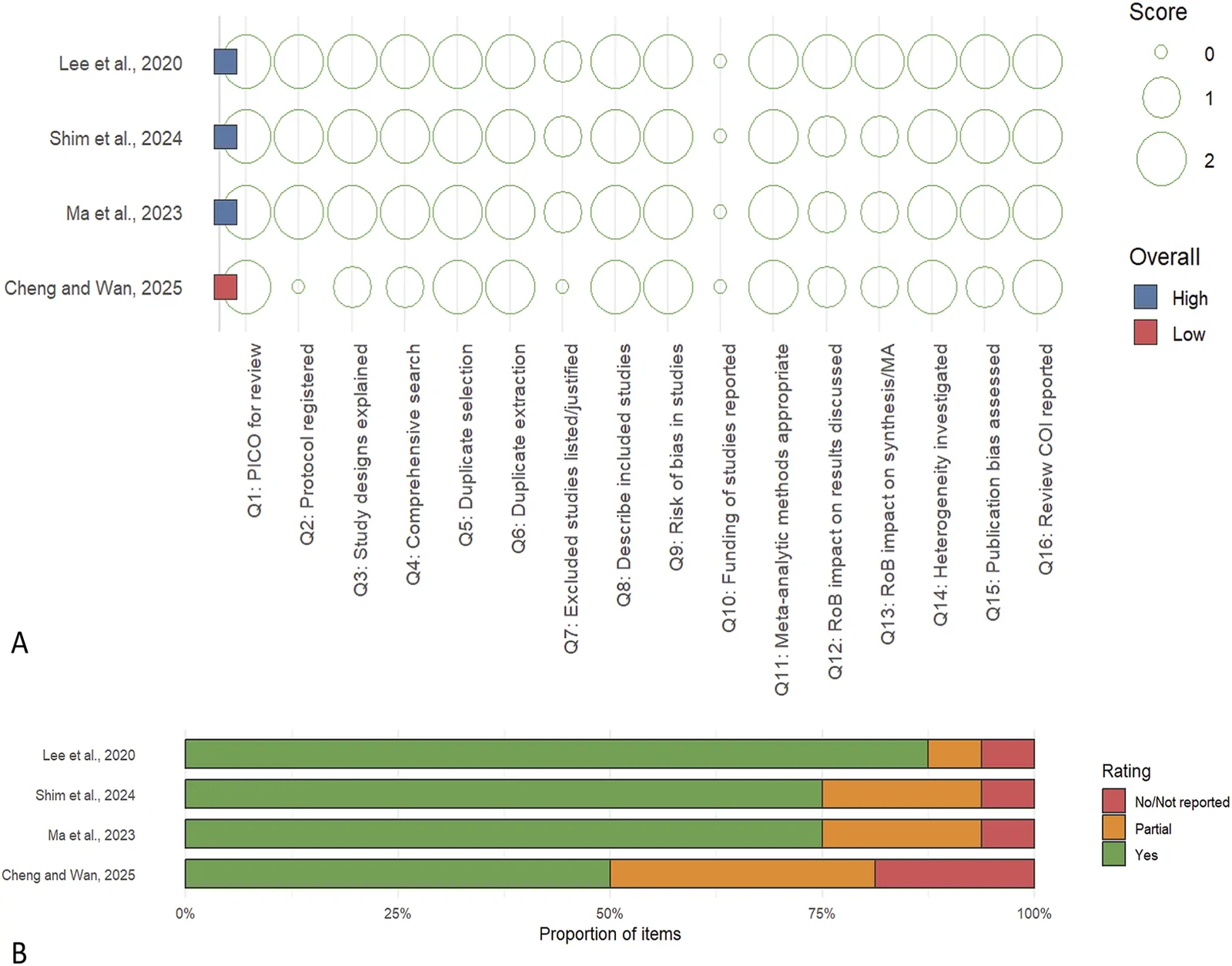
AMSTAR-2 methodological appraisal of included systematic reviews/meta-analyses. **(A)** Item-level AMSTAR-2 judgments by review. **(B)** Distribution of item-level responses. Overall confidence was classified using standard AMSTAR-2 categories only: high, moderate, low, or critically low.

The methodological quality of the four included systematic reviews/meta-analyses was assessed using the AMSTAR-2 framework (Shea et al., 2017) (Figure 2). We used only the standard AMSTAR-2 overall confidence categories: high, moderate, low, or critically low. Hybrid labels such as “low–moderate” and ““moderate–high” were removed. Lee et al. (2020) was rated as high because most critical AMSTAR-2 domains were fulfilled, the review was prospectively registered, the search was comprehensive, the risk of bias was assessed, and GRADE evidence tables were presented. Ma et al. (2023) was rated as moderate because it had protocol registration, broad database coverage, risk-of-bias assessment, subgroup/sensitivity analyses, and publication-bias testing but lacked full GRADE assessment and had incomplete reporting of primary-study funding. Shim et al. (2024) was rated as moderate because it used a comprehensive search strategy and network meta-analysis methods and assessed the risk of bias but did not provide GRADE certainty assessments and had incomplete reporting of funding sources/conflicts of interest for the included trials. Cheng and Wan (2025) was rated as low because preregistration/protocol information, excluded-study transparency, and funding reporting were incomplete. These AMSTAR-2 ratings reflect confidence in the conduct of the systematic reviews, not the certainty of clinical effects. The certainty of clinical findings was considered separately at the outcome level.

### 3.4 Findings by outcome

#### 3.4.1 Clinical efficacy rate

The outcome labeled as “clinical efficacy rate” was not uniformly defined across the primary trials and often represented a trial-defined composite response based on symptoms, secondary sexual characteristics, hormone levels, or ultrasound indices. Therefore, we interpreted this outcome as a non-standardized composite response rather than a uniform clinical endpoint. Across the included reviews, selected botanical drug preparations or combined regimens showed favorable signals for this composite response, but the certainty was low due to subjective definitions, unclear blinding, small trial sizes, and inconsistent reporting. Lee et al. (2020) found that HM significantly increased response rates compared with controls (RR = 1.14), particularly when combined with megestrol, while comparisons with triptorelin showed higher but non-significant efficacy. Ma et al. (2023) reported that NYPF therapy, alone or combined with GnRHa, improved the trial-defined clinical efficacy rate compared with GnRHa (RR = 1.15, 95% CI = 1.11–1.20; I^2^ = 39%; 20 RCTs, 1,829 participants), although this endpoint was not uniformly defined across trials. Cheng and Wan (2025) further highlighted that CHM + GnRHa consistently improved clinical responses, especially for Zhibai Dihuang-based regimens. Shim et al. (2024), in a large Bayesian network meta-analysis, demonstrated that combinations such as HM + GnRHa or HM + auricular plaster therapy generally ranked higher for response than monotherapies.

#### 3.4.2 Secondary sexual characteristics

Significant reductions in uterine and ovarian volume were consistently observed across reviews, although results varied by formula. Cheng and Wan (2025) reported that CHM + GnRHa significantly decreased uterine volume (SMD = −0.97, 95% CI = −1.34 to −0.61; I^2^ = 89%; 13 RCTs, 1,174 participants) and ovarian volume (SMD = −0.79, 95% CI = −1.15 to −0.42; I^2^ = 90%; 13 RCTs, 1,272 participants), although both estimates showed high heterogeneity. Lee et al. (2020) found smaller but significant reductions in uterine volume (SMD = −0.58) and non-significant reductions in ovarian volume. Ma et al. (2023) confirmed that HM lowered uterine volume (MD = −0.70), but not ovarian volume. Shim et al. (2024) ranked megestrol as most effective for uterine volume, while HM + auricular plaster therapy was optimal for ovarian volume. Evidence for breast development was more limited; Lee et al. (2020) and Shim et al. (2024) both noted potential delays in maturation with HM- or KM-based combinations, although findings were inconsistent.

#### 3.4.3 Hormone levels

All four reviews showed consistent reductions in sex hormones with HM interventions. Cheng and Wan (2025) demonstrated robust decreases in FSH (SMD = −1.84), LH (SMD = −2.07), and E2 (SMD = −1.49), with stronger effects from Zhibai Dihuang than from Dabuyin. Lee et al. (2020) reported lower estradiol for HM than for the control group using mean difference units (MD = −4.22 pg/mL, 95% CI = −5.00 to −3.45; I^2^ = 0%; 7 RCTs, 464 participants) and also found lower LH and E2 for HM than for triptorelin. Ma et al. (2023) reported lower sex hormone levels for NYPF therapy ± GnRHa than for GnRHa, including FSH (SMD = −1.56, 95% CI = −2.06 to −1.06; I^2^ = 96%), LH (SMD = −1.52, 95% CI = −2.07 to −0.98; I^2^ = 96%), and E2 (SMD = −1.09, 95% CI = −1.46 to −0.72; I^2^ = 93%). Shim et al. (2024), through network ranking, showed that HM + GnRHa was the most effective intervention for reducing LH and E2, while HM + auricular plaster therapy + GnRHa was the most effective for FSH, consistently outperforming GnRHa monotherapy.

#### 3.4.4 Bone age index

Evidence on skeletal maturation was mixed but generally trended toward a benefit with HM. Cheng and Wan (2025) found that CHM + GnRHa significantly reduced BAI compared with GnRHa alone (SMD = −1.69, 95% CI = −2.58 to −0.80; I^2^ = 97%; 10 RCTs, 847 participants), but the very high heterogeneity and surrogate nature of BAI limit clinical certainty. Lee et al. (2020) reported a significant reduction in BAI compared with controls (SMD = −0.64) and significant improvement when HM was combined with megestrol (MD = −0.68). Ma et al. (2023) also showed that HM significantly lowered BAI overall (MD = −0.35). In the network analysis by Shim et al. (2024), HM + auricular plaster therapy ranked the highest for BAI improvement (SUCRA 86.8%), followed by HM + megestrol and HM + GnRHa.

#### 3.4.5 Adverse events

Reported adverse events included gastrointestinal discomfort, nausea, vomiting, rash, injection-site pain, transient vaginal bleeding, appetite changes, and occasional liver enzyme abnormalities. No serious adverse events were reported in the included reviews. However, this should not be interpreted as proof of safety. Adverse-event definitions, severity grading, active surveillance methods, withdrawal criteria, hepatic/renal monitoring, and duration of follow-up were inconsistently reported. Because the target population is pediatric and the interventions may be used during a sensitive endocrine-development window, the current evidence is insufficient to determine long-term safety, including effects on growth velocity, bone maturation, final adult height, menarche, ovulatory function, metabolic outcomes, hepatic and renal function, allergic reactions, product contamination, adulteration, or interactions with GnRHa or megestrol.

#### 3.4.6 Subgroup findings

Subgroup analyses converged on Zhibai Dihuang as the most consistent formula, showing strong effects on reducing hormone levels and pelvic ultrasound measures. The Dabuyin pill also reduced hormone levels but had less reliable effects on uterine/ovarian volume and BAI. NYPF regimens, more explicitly reported in the classification by Ma et al. (2023) and Shim et al. (2024), showed positive but variable results, with newer formulations ranking the highest for BAI, LH, FSH, and E2. Across reviews, botanical drug preparations combined with GnRHa showed favorable adjunctive signals compared with GnRHa alone. However, these findings should be considered hypothesis-generating because formulation composition, dosing, comparator choice, outcome definitions, and risk of bias varied across the underlying trials.

To improve clinical interpretability and avoid overstating the statistical findings, the principal outcomes were summarized according to their reported effect measures, measurement units, clinical significance, and certainty concerns. Because several included reviews reported standardized mean differences or used inconsistent outcome definitions, these estimates should be interpreted as directional signals rather than direct clinical units unless a mean difference with a clearly reported unit was available (Table 3).

**TABLE 3:** Clinical interpretation of effect measures, outcome units, and certainty concerns across major CPP/ICPP outcomes.

Outcome	Effect measure and unit	Clinical interpretation	Certainty/concern
Clinical response	RR; unitless	Indicates relative probability of meeting trial-defined response criteria; definitions varied across trials	Low/very low; subjective definitions and unclear blinding
Uterine volume	MD in mL/cm^3^ when reported; SMD unitless	Smaller uterine volume may reflect reduced estrogenic stimulation, but measurement protocols varied	Low; ultrasound methods and timing inconsistent
Ovarian volume	MD in mL/cm^3^ when reported; SMD unitless	May reflect pubertal suppression, but ovarian volume is variable and method-dependent	Low/very low
FSH/LH	IU/L or mIU/mL when MD; SMD unitless	Lower gonadotropins may suggest HPG-axis suppression, but basal vs. stimulated values were inconsistently reported	Low/very low
Estradiol	pg/mL or pmol/L when MD; SMD unitless	Lower estradiol may indicate reduced ovarian steroidogenesis; timing and assay methods varied	Low/very low
Bone age index	Usually unitless ratio or index	Lower BAI may suggest slower skeletal maturation; final adult height was rarely assessed	Low/very low
Adverse events	Counts, RR, or narrative	No serious events were reported, but reporting was incomplete and under-reporting is possible	Very low safety certainty

Abbreviations: AE, adverse event; BAI, bone age index; CPP, central precocious puberty; E2, estradiol; FSH, follicle-stimulating hormone; GnRHa, gonadotropin-releasing hormone agonist; HPG, hypothalamic–pituitary–gonadal; ICPP, idiopathic central precocious puberty; LH, luteinizing hormone; MD, mean difference; RR, risk ratio; SMD, standardized mean difference. MD values retain the original measurement unit reported by the included review; SMD values are unitless and should not be interpreted as absolute clinical changes.

To provide a complete quantitative summary beyond the clinical interpretation of effect measures, Table 4 reports the main pooled or network estimates extracted from the included reviews, including the comparison, number of contributing RCTs and participants, effect estimate with 95% CI where available, heterogeneity, and certainty judgment.

**TABLE 4:** Summary of main effect estimates and certainty judgments for major CPP/ICPP outcomes.

Outcome	Comparison	Source review	No. of RCTs	Participants	Effect estimate	95% CI	Heterogeneity	Certainty judgment	Notes
Clinical efficacy rate	HM vs. control	Lee et al. (2020)	6	418	RR 1.14	1.03 to 1.26	I^2^ = 2%	Moderate, reported GRADE	Trial-defined composite response; definitions varied across trials
Clinical efficacy rate	NYPF therapy ± GnRHa vs. GnRHa	Ma et al. (2023)	20	1,829	RR 1.15	1.11 to 1.20	I^2^ = 39%	Low, author-derived	Not uniformly defined; no formal GRADE reported
Uterine volume	CHM + GnRHa vs. GnRHa	Cheng and Wan (2025)	13	1,174	SMD −0.97	−1.34 to −0.61	I^2^ = 89%	Very low, author-derived	SMD is unitless; high heterogeneity
Ovarian volume	CHM + GnRHa vs. GnRHa	Cheng and Wan (2025)	13	1,272	SMD −0.79	−1.15 to −0.42	I^2^ = 90%	Very low, author-derived	SMD is unitless; high heterogeneity
FSH	CHM + GnRHa vs. GnRHa	Cheng and Wan (2025)	15	1,358	SMD −1.84	−2.33 to −1.36	I^2^ = 93%	Very low, author-derived	SMD is unitless; serum sampling methods were not consistently standardized
LH	CHM + GnRHa vs. GnRHa	Cheng and Wan (2025)	15	1,358	SMD −2.07	−2.78 to −1.37	I^2^ = 96%	Very low, author-derived	SMD is unitless; high heterogeneity
Estradiol	CHM + GnRHa vs. GnRHa	Cheng and Wan (2025)	15	1,358	SMD −1.49	−1.89 to −0.94	I^2^ = 92%	Very low, author-derived	SMD is unitless; assay timing and methods varied
Estradiol	HM vs. control	Lee et al. (2020)	7	464	MD −4.22 pg/mL	−5.00 to −3.45	I^2^ = 0%	Moderate, reported GRADE	MD retains the original unit reported by the review
Bone age index	CHM + GnRHa vs. GnRHa	Cheng and Wan (2025)	10	847	SMD −1.69	−2.58 to −0.80	I^2^ = 97%	Very low, author-derived	Surrogate outcome; final adult height rarely assessed
Bone age index	HM + auricular plaster therapy ranked against other interventions	Shim et al. (2024)	31	NR	SUCRA 86.8%	Not applicable	No problematic inconsistency reported for the BAI network	Very low, author-derived	Ranking probability only; not an absolute treatment effect
Adverse events	NYPF therapy ± GnRHa vs. GnRHa	Ma et al. (2023)	10 reporting RCTs	1,187	RR 0.62	0.36 to 1.08	I^2^ = 0%	Very low, author-derived	No statistically significant difference; under-reporting remains possible

Abbreviations: AE, adverse event; BAI, bone age index; CHM, Chinese herbal medicine; CI, confidence interval; CPP, central precocious puberty; E2, estradiol; FSH, follicle-stimulating hormone; GnRHa, gonadotropin-releasing hormone agonist; GRADE, Grading of Recommendations Assessment, Development, and Evaluation; HM, herbal medicine; ICPP, idiopathic central precocious puberty; LH, luteinizing hormone; MD, mean difference; NR, not reported; NYPF, Nourishing Yin–Purging Fire; RCT, randomized controlled trial; RR, risk ratio; SMD, standardized mean difference; SUCRA, surface under the cumulative ranking curve. Certainty judgments are formal GRADE ratings only where reported by the included review; otherwise, they represent author-derived umbrella-review certainty judgments based on risk of bias, inconsistency, indirectness, imprecision, publication bias, and overlap of primary trials.

### 3.5 Overlap between reviews

**FIGURE 3:**
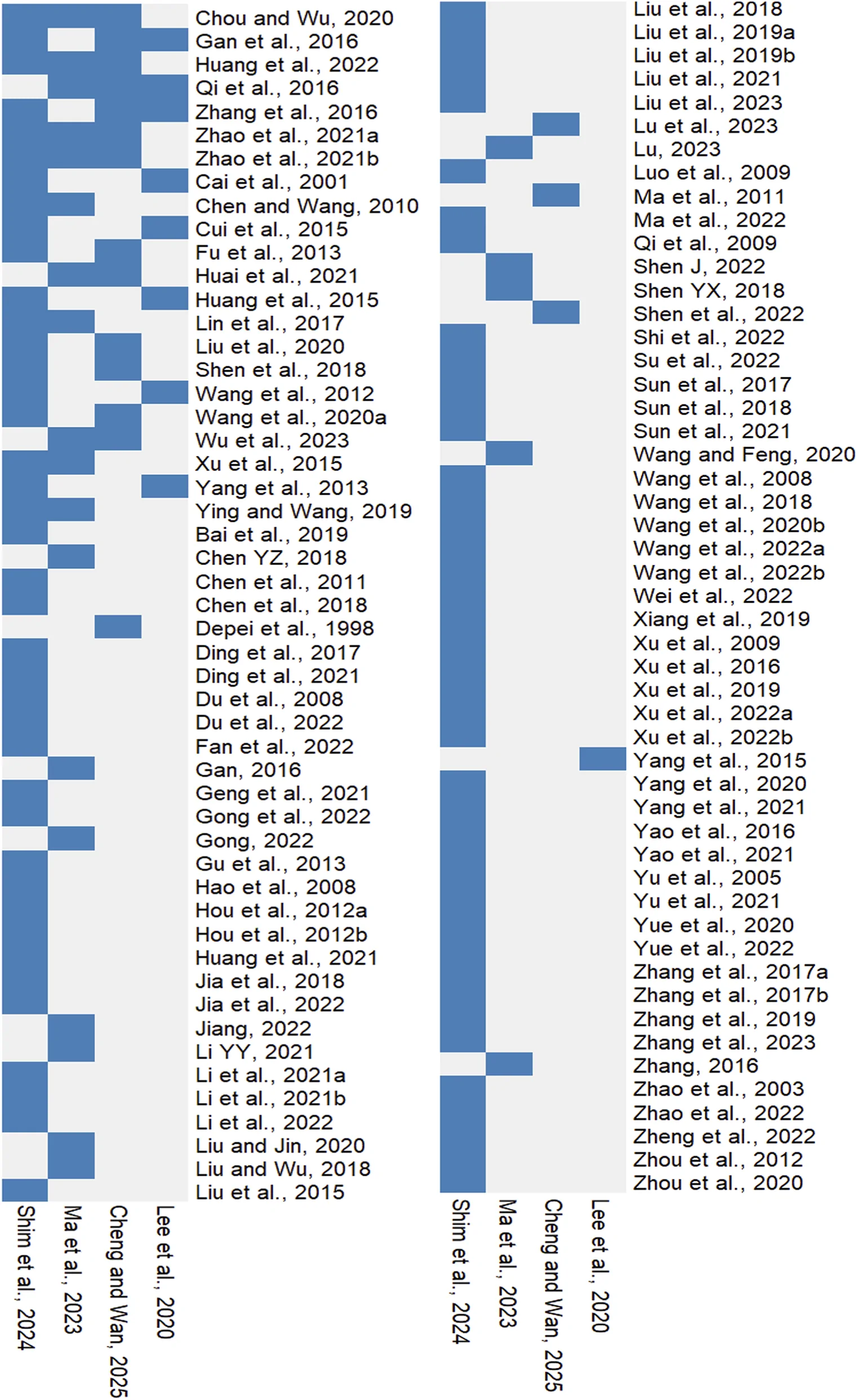
Overlap of primary randomized controlled trials across systematic reviews/meta-analyses on botanical drug preparations and related East Asian medicine interventions for CPP/ICPP.

Using the CCA approach described by Pieper et al. (2014), the citation matrix identified 102 unique primary trials across the four included reviews, with 131 primary-study occurrences (Figure 3). The overall corrected covered area was 9.5%, indicating moderate overlap. Pairwise CCA was the highest between Ma et al. (2023) and Cheng and Wan (2025) at approximately 21.2%, indicating very high overlap, followed by Cheng and Wan (2025) with Lee et al. (2020) at approximately 13.0% and Shim et al. (2024) with Cheng and Wan (2025) at approximately 11.2%, indicating high overlap. Lower pairwise overlap was observed between Ma et al. (2023) and Lee et al. (2020) at approximately 3.2%. These findings indicate that some apparently consistent conclusions are partly based on duplicated primary trials rather than completely independent evidence.

## 4 Discussion

This umbrella review found favorable but low-certainty signals for selected East Asian botanical drug preparations in CPP/ICPP, particularly when used as adjuncts to GnRHas. However, these findings should be interpreted cautiously. The evidence does not support the generalized conclusion that “TCM,” as a broad category, is effective for CPP/ICPP. Rather, the available data relate to heterogeneous multi-component preparations, including Zhibai Dihuang-based formulas, Dabuyin-based formulas, and broader Nourishing Yin–Purging Fire regimens, with variable compositions, doses, comparators, and treatment durations. Because most primary trials were small, single-center studies with unclear randomization, limited allocation concealment, lack of blinding, and inconsistent safety reporting, the observed effects should be considered hypothesis-generating rather than confirmatory.

The direction of reported effects—lower pelvic ultrasound indices, lower gonadotropin/estradiol measures, and slower skeletal maturation—was compatible with CPP treatment goals. However, because the evidence was heterogeneous and at low certainty, these findings do not prove additive physiological control or establish clinical effectiveness. Whether these surrogate changes translate into improved final adult height (FAH), reproductive outcomes, metabolic health, or psychosocial outcomes remains uncertain. Although FAH was not directly pooled in these reviews, improvements in BAI and pelvic indices are encouraging surrogate signals; still, long-term outcomes remain insufficiently studied. From a regimen standpoint, Zhibai Dihuang consistently emerged as a useful backbone, while NYPF formulations (with the Zhimu–Huangbai pair) provide a mechanistic bridge—linking clinical signals to plausible endocrine and anti-inflammatory pathways highlighted in network pharmacology.

Across reviews, AEs with CHM were infrequent and mild. Notably, vaginal bleeding was reported in the GnRHa arms but not in the CHM + GnRHa arms in at least one trial set; pooled network comparisons showed fewer AEs with CHM alone than with GnRHa and fewer AEs with CHM + GnRHa than with GnRHa alone, suggesting a potential mitigating effect on some GnRHa-related side effects. Nevertheless, pharmacovigilance remains essential given the heterogeneous herbal compositions, dosing regimens, and reporting practices.

Despite convergent benefits, all four reviews documented important risks of bias in the underlying RCTs: small samples, single-center Chinese trials, inconsistent randomization/concealment, absence of blinding/placebos, and high heterogeneity. Only one review systematically applied GRADE to summarize certainty (Lee et al., 2020), while others did not, limiting confidence in pooled magnitudes. The network meta-analysis offered comparative rankings (SUCRA) but still relied on many small studies, leaving room for small-study effects and residual confounding (Curteis et al., 2025).

Another important limitation is the geographic and cultural concentration of the evidence. Nearly all primary trials were conducted in China, and most reported favorable results. This pattern raises concerns about selective publication, language bias, small-study effects, and contextual factors that may not generalize to other healthcare systems or regulatory settings. Because this umbrella review included English-language systematic reviews and did not reanalyze unpublished trial data, the magnitude of benefit may be overestimated.

Generalizability is limited by near-exclusive conduct in China, variability in herbal identities (decoction/pill/granule), dosing, and co-interventions. Standardization of hormone sampling (basal vs. peak; luteal vs. follicular timing), ultrasound protocols, and BAI definitions was frequently unclear, contributing to heterogeneity. Our overlap analysis demonstrated substantial reuse of the same RCTs across reviews (especially around Zhibai/Dabuyin families), emphasizing the need to account for double-counting when interpreting aggregated signals.

This umbrella review has several strengths. It synthesizes contemporary systematic reviews and meta-analyses, separates botanical drug preparations from related non-botanical East Asian medicine modalities, maps intervention heterogeneity, applies AMSTAR-2 and ROBIS at the review level, summarizes certainty using GRADE-informed domains, and quantifies overlap using corrected covered area. However, several limitations remain important. The search was limited to PubMed/MEDLINE, Web of Science, and Scopus and to English-language SRs/MAs. Chinese and Korean databases were not searched at the umbrella-review level, which may have resulted in the omission of relevant non-English evidence syntheses. In addition, we relied on review-level extraction rather than reanalysis of all primary trials, and several outcomes were affected by overlap, inconsistent definitions, incomplete safety reporting, and low methodological quality of the underlying RCTs.

This umbrella review includes several limitations. First, we restricted the review to English-language SRs/MAs and did not include non-English syntheses, which may have biased evidence coverage. Second, we relied on review-level extractions rather than re-meta-analyzing individual trials; thus, we inherited each review’s limitations (risk-of-bias judgments, analytic choices, and reporting). Third, although we mapped overlap and emphasized narrative synthesis, we did not quantitatively adjust outcome estimates to account for cross-review duplication. Finally, certainty grading was as-reported (only one review provided full GRADE tables), so we did not harmonize certainty ratings across outcomes.

For future research, high-quality, multicenter RCTs outside of China are urgently needed to test CHM + GnRHa vs. GnRHa alone with prespecified protocols, adequate allocation concealment, and placebo/attention controls where feasible. Core outcomes should include BAI, pelvic ultrasound indices, hormone panels with standardized timing, and, critically, long-term endpoints: FAH, menarche/ovulatory function, metabolic outcomes, and psychological outcomes. Furthermore, formulation standardization and quality control must progress beyond botanical names, accompanied by dose-finding studies and pragmatic comparative-effectiveness trials to clarify which formula backbones and which combination strategies offer the best benefit–risk profiles for specific patient populations. For future RCTs, it is necessary to link network pharmacology hypotheses to clinical biomarkers (kisspeptin, GnRH pulsatility proxies, and ovarian steroidogenesis) in translational trials to validate these pathways beyond *in silico* inference.

Mechanistic interpretation remains preliminary. Some included reviews discussed putative plant metabolites such as quercetin, kaempferol, β-sitosterol, or berberine-containing botanical drug materials and proposed endocrine or anti-inflammatory pathways. However, these hypotheses were largely derived from network pharmacology or indirect pharmacological evidence rather than pediatric CPP biomarker studies. Therefore, they should be interpreted as mechanistic hypotheses rather than validated mechanisms of clinical efficacy. Future trials should connect formulation chemistry and quality control with endocrine biomarkers, including basal and stimulated LH/FSH, estradiol, kisspeptin-related pathways, ovarian steroidogenesis markers, growth velocity, and bone-age progression.

## 5 Conclusion

The available systematic reviews and meta-analyses suggest a possible adjunctive benefit of selected botanical drug preparations, particularly Zhibai Dihuang-based and related Nourishing Yin–Purging Fire regimens used with GnRHa, for children with CPP/ICPP. However, certainty remains low because of heterogeneous formulations, incomplete species-level reporting, variable comparators, overlapping primary trials, unclear or high risk of bias, single-country evidence, incomplete database coverage at the umbrella-review level, and inadequate long-term pediatric safety data. Current evidence is insufficient to establish these preparations or related East Asian medicine modalities as validated standalone or routine therapeutic strategies. Future multicenter randomized trials should use standardized botanical authentication, dose reporting, comparator selection, endocrine outcome definitions, active safety monitoring, and long-term follow-up, including final adult height, reproductive outcomes, metabolic safety, and psychosocial outcomes.

## Data Availability

The raw data supporting the conclusions of this article will be made available by the authors, without undue reservation.
