# Supplementary material for: Botanical drug preparations and related East Asian medicine interventions for central and idiopathic central precocious puberty: an umbrella review of systematic reviews and meta-analyses
Source: Front Pharmacol. 2026 Aug 19;17:1697393. doi: 10.3389/fphar.2026.1697393 (PMC13533962; doi:10.3389/fphar.2026.1697393)
Supplement: Supplementary file 1 [file Table1.docx]

Table S1. Taxonomic validation and composition reporting of the main botanical drug preparations and non-botanical drug materials evaluated in the included reviews

| Formula / preparation | Drug material / pinyin | Validated scientific name | Family | Drug name / plant part | Reported in original evidence? |
| --- | --- | --- | --- | --- | --- |
| Zhibai Dihuang Wan | Zhi Mu | *Anemarrhena asphodeloides* Bunge | Asparagaceae | Anemarrhenae Rhizoma | Yes / NR |
| Zhibai Dihuang Wan / Dabuyin | Huang Bai | *Phellodendron chinense* C.K.Schneid. or *Phellodendron amurense* Rupr. | Rutaceae | Phellodendri Cortex | Yes / NR |
| Zhibai Dihuang Wan / Dabuyin | Shu Di Huang | *Rehmannia glutinosa* (Gaertn.) Libosch. ex DC. | Orobanchaceae | Rehmanniae Radix Praeparata | Yes / NR |
| Zhibai Dihuang Wan | Shan Zhu Yu | *Cornus officinalis* Siebold & Zucc. | Cornaceae | Corni Fructus | Yes / NR |
| Zhibai Dihuang Wan | Shan Yao | *Dioscorea polystachya* Turcz. | Dioscoreaceae | Dioscoreae Rhizoma | Yes / NR |
| Zhibai Dihuang Wan | Ze Xie | *Alisma plantago-aquatica* subsp. *orientale* (Sam.) Sam. / *Alisma orientale* (Sam.) Juz. | Alismataceae | Alismatis Rhizoma | Yes / NR |
| Zhibai Dihuang Wan | Mu Dan Pi | *Paeonia × suffruticosa* Andrews | Paeoniaceae | Moutan Cortex | Yes / NR |
| Zhibai Dihuang Wan | Fu Ling | *Wolfiporia cocos* / *Poria cocos* naming convention | Polyporaceae | Poria, sclerotium | Yes / NR |
| Dabuyin Wan | Gui Ban | Non-botanical animal-derived drug | — | Testudinis Carapax et Plastrum | Yes / NR |

Abbreviations: NR, not reported. Scientific names and families should be verified against recognized taxonomic databases before final submission. Non-botanical materials, such as Gui Ban, are listed separately and were not treated as plant-derived botanical drug materials.
